# Effects of l-Serine on Macrolide Resistance in Streptococcus suis

**DOI:** 10.1128/spectrum.00689-22

**Published:** 2022-07-18

**Authors:** Tong Wu, Xiaozhen Wang, Yue Dong, Chen Xing, Xueying Chen, Lu Li, Chunliu Dong, Yanhua Li

**Affiliations:** a College of Veterinary Medicine, Northeast Agricultural University, Harbin, Heilongjiang, China; b Heilongjiang Key Laboratory for Animal Disease Control and Pharmaceutical Development, Harbin, Heilongjiang, China; University of Florida College of Dentistry

**Keywords:** bacterial resistance, *Streptococcus suis*, l-serine, metabolism, macrolides, ROS, DNA damage

## Abstract

Streptococcus suis is an important zoonotic pathogen. Due to the indiscriminate use of macrolides, S. suis has developed a high level of drug resistance, which has led to a serious threat to human and animal health. However, it takes a long time to develop new antibacterial drugs. Therefore, we consider the perspective of bacterial physiological metabolism to ensure that the development of bacterial resistance to existing drugs is alleviated and bacterial susceptibility to drugs is restored. In the present study, an untargeted metabolomics analysis showed that the serine catabolic pathway was inhibited in drug-resistant S. suis. The addition of l-serine restored the fungicidal effect of macrolides on S. suis *in vivo* and *in vitro* by enhancing the serine metabolic pathway. Further studies showed that l-serine, stimulated by its serine catabolic pathway, inhibited intracellular H_2_S production, reduced Fe-S cluster production, and restored the normal occurrence of the Fenton reaction in cells. It also attenuated the production of glutathione, an important marker of the intracellular oxidation-reduction reaction. All these phenomena eventually contribute to an increase in the level of reactive oxygen species, which leads to intracellular DNA damage and bacterial death. Our study provides a potential new approach for the treatment of diseases caused by drug-resistant S. suis.

**IMPORTANCE** The emergence of antimicrobial resistance is a global challenge. However, new drug development efforts consume considerable resources and time, and alleviating the pressure on existing drugs is the focus of our work. We investigated the mechanism of action of l-serine supplementation in restoring the use of macrolides in S. suis, based on the role of the serine catabolic pathway on reactive oxygen species levels and oxidative stress in S. suis. This pathway provides a theoretical basis for the rational use of macrolides in clinical practice and also identifies a possible target for restoring drug sensitivity in S. suis.

## INTRODUCTION

Antibiotics have been used to treat microbial infections in humans and animals (1). However, the overuse of antibiotics has caused bacteria to exhibit different levels of resistance (2). Meanwhile, the development of new antibiotics has been quite slow (3), and new strategies are needed to improve the effectiveness of existing antibiotics to cope with emerging antibiotic resistance (4). With the development of modern techniques, such as omics and systems biology, it is becoming clear that the emergence of drug resistance is often accompanied by changes in metabolic pathways within bacteria (5). Changes in some by-products of bacterial metabolic pathways may promote bacterial resistance to multiple antimicrobial drugs (6). (Bacterial resistance is a phenomenon in which bacteria are no longer sensitive to antibacterial drugs, making clinical drugs less effective, and is a form of bacterial adaptation to the environment.) Even this alteration can lead to an increase in persistent bacteria that induce infection *in vivo* and enhanced biofilm formation (7). (Persistent bacteria are a subgroup of dormant bacteria that are resistant to antimicrobial drugs and can be revived under certain conditions, and as such they are an important cause of recurrent and chronic infections).

Amino acids are important for bacterial growth and metabolism of bacteria (8). The acquisition of amino acids is a fundamental function of all cellular physiological metabolism. Amino acids and peptides in the culture medium can be taken up directly by bacteria for their own catabolism or biosynthesis, and this maintains the dynamic balance of amino acids in bacteria and resists exogenous stimuli (9). The emergence of specific features in the physiological metabolism of bacteria is associated with antibiotic resistance and alters the relative susceptibility of bacteria to antibiotic drugs. Bacteria with partially reduced metabolic pathways have been shown to be resistant or tolerant to a wide range of antibiotics, and increased drug susceptibility has been associated with enhanced metabolism (10). This has led to intensive research on amino acids as antimicrobial adjuvants to better eradicate drug-resistant or persistent bacteria (11). The addition of exogenous metabolites (e.g., glucose or malate) to restore metabolic defects and treat drug-resistant pathogens in combination with otherwise-ineffective antibiotics is an attractive approach (12, 13). However, there are few studies on whether Streptococcus suis resistance is associated with changes in physiological metabolism.

Altered amino acid metabolic pathways induced by exogenous amino acid supplementation in Escherichia coli (14) and Mycobacterium spp. (15) have been shown to affect oxidative stress and enhance endogenous reactive oxygen species (ROS) production in bacteria. ROS are key substances in balancing oxidative stress that occurs in bacteria (16). The addition of exogenous metabolites can alter the physiological metabolism of bacteria and affect the level of ROS. Oxidative stress occurs when ROS accumulate to a certain level in bacteria and the produced hydroxyl radicals cause oxidative damage to cells by interacting with cellular components (lipids, DNA, and proteins) (17). Enhanced uptake of a drug by bacteria contributes to the induction of DNA damage, and the bactericidal effect of the drug on drug-resistant strains is subsequently enhanced (15). Therefore, we propose that exogenous metabolites may reduce drug tolerance in resistant strains if they promote bacterial DNA damage by increasing endogenous ROS levels. S. suis is a major porcine bacterial pathogen that can also be transmitted to humans through the invasion of mucous membranes and wounds (18), leading to infection in people who work with pigs and thereby posing a great threat to the development of the pig industry and to public health safety (19). One of the main causes of persistent S. suis infections is the clinical isolation of strains with varying degrees of resistance to drugs, including macrolides, fluoroquinolones, and tetracyclines (20). We investigated whether exogenous metabolites have the potential to restore the sensitivity of S. suis to macrolides by altering their physiological metabolic profiles. Here, we describe in detail the metabolic state of S. suis strains resistant to macrolide antibiotics and demonstrate that l-serine affects the serine catabolic pathway of S. suis and, in the presence of metabolites, stimulates ROS production and restores the susceptibility of this bacterium to macrolides.

## RESULTS

### Nontargeted HPLC-MS/MS metabolic profile analysis.

**FIG 1:**
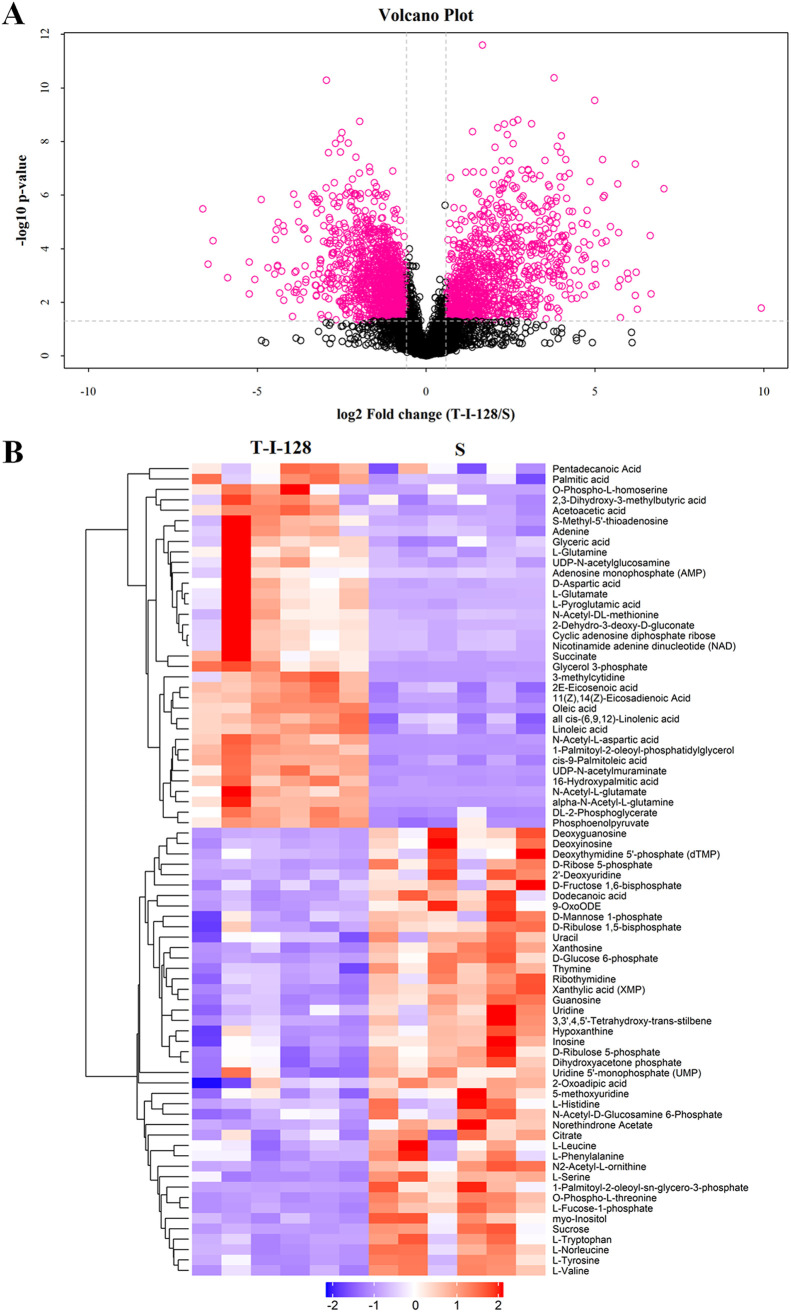
Metabolic profiling and bioinformatic analysis of drug-sensitive (S) and drug-resistant (T-I-128) strains. (A) A volcano plot demonstrated metabolites altered significantly are from comparisons of resistant strain and sensitive strain groups. The volcano plot is a combination of the fold change (S/T-I-128) and *t* tests, with the *x* axis indicating log_2_ fold change [log_2_(FC)] and the *y* axis showing the −log_10_(*P* value). Each dot represents one metabolite, with the pink dot metabolite indicating that they have passed the threshold for both the *t* test (*P *< 0.01) and have an FC of > 2. (B) Overview metabolic profile heatmap showing the comparison of the control group sensitive strain to the resistant strain in this study. Six biological replicates from the control group and six biological replicates from the resistant strain group are plotted on this heatmap.

We performed metabolomic analysis of a macrolide-resistant strain (T-I-128) with a macrolide resistance gene and the sensitive strain ATCC 700494 (S) by high-performance liquid chromatography–mass spectrometry (HPLC-MS). The analytical performance was evaluated by injecting multiple quality control (QC) samples between the biological samples, and the correlation coefficient of the QC samples during the entire MS run was calculated to be 0.98, which suggested that our analytical method is robust and reproducible. Each tested bacterial group contained six biological replicates to ensure reproducibility. Unsupervised principal component analysis (PCA) (see Fig. S1 in the supplemental material) and orthogonal partial least-squares discriminant analysis (OPLS-DA) (see Fig. S2) were used to evaluate the groups. The fractions of R2Y (the square of the percentage of original data information retained on the *x* axis) and Q2 (the prediction rate of the model) in the statistical analysis were both greater than 0.5 (see Table S2), proving that our model is reliably established, highly reproducible, and has good stability. To further decipher the metabolic differences between the three comparison groups, a combined comparison of metabolite *t* test, *P* value, and fold change (FC) of control samples versus resistant strain samples was conducted. These results were used to generate volcano plots. In the volcano plot of Fig. 1A, pink dots represent those metabolites that passed both the *t* test (*P *< 0.05), fold change (0.667 < FC > 1.5), and model variable weights (variable influence of projection [VIP] of >1) threshold. There were 78 different metabolites for the sensitive and resistant strains (Fig. 1B; see also Table S3). Among them, the ploidy change in l-serine was found to be upregulated in the differential metabolites. In parallel, the strongly affected KEGG (Kyoto Encyclopedia of Genes and Genomes) pathway was that for metabolism of glycine, serine, and threonine metabolism (see Fig. S3). We observed that glycine, serine, and threonine metabolism had a strong effect on l-serine. l-Serine plays an important regulatory role in the biosynthesis of glycine (21) and cysteine (22), respectively. It has been shown that glycine and cysteine can affect drug-resistant bacteria in different ways (23, 24). These results prompted us to explore whether the phenotype of S. suis changed in the presence of l-serine.

### l-Serine increased the susceptibility of planktonic S. suis cells to macrolide antibiotics.

**FIG 2:**
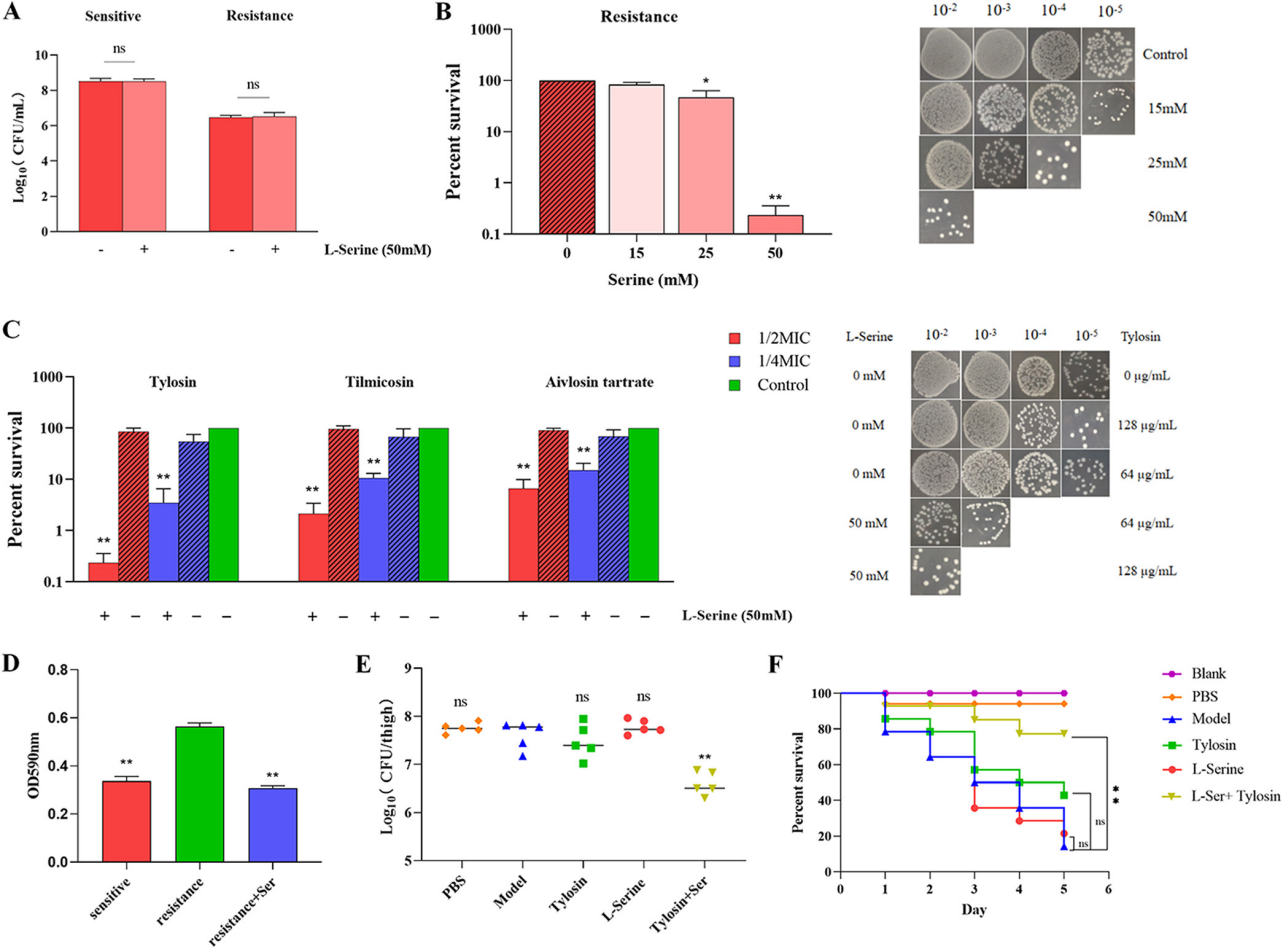
Effect of l-serine addition on the survival and biofilm formation of S. suis in *in vitro* culture and on the treatment of S. suis after host infection *in vivo*. (A) Effect of adding 50 mM l-serine on the survival of S. suis. (B) Effect of 15, 25, and 50 mM concentrations of l-serine in combination with 64 mg/L tylosin (1/2 MIC) on the survival of drug-resistant strains. (C) Synergistic effect of 50 mM l-serine in combination with macrolides on bacterial survival. Tylosin concentration: 1/2 MIC (64 mg/L) or 1/4 MIC (32 mg/L); tilmicosin concentration: 1/2 MIC (16 mg/L) or 1/4 MIC (8 mg/L); aivlosin tartrate concentration: 1/2 MIC (128 mg/L) or 1/4 MIC (64 mg/L). (D) Effect of 50 mM l-serine addition on biofilm formation ability of S. suis. (E) Therapeutic effects of tylosin and l-serine alone and in combination in neutrophilic mice infected with drug-resistant strains. The bacterial load of infected thigh muscle in neutropenic mice (*n* = 5 per group) was determined by colony counting. Model: The mice with persistent 24h infection after intramuscular injection of drug-resistant bacteria into the thigh. All data are the means ± s.d. *P* values were determined using an unpaired, two-tailed Student's *t* test. ns, *P* > 0.05; *, *P *< 0.05; **, *P* < 0.01. (F) Survival of *G. mellonella* larvae injected with 10^7^ CFU/mL of resistant strain and after treatment with l-serine and tylosin alone or in combination. In this model, persistent infection of *G. mellonella* occurred within 5 days after injection of drug-resistant bacteria at the right posterior gastropod. The survival curves were compared based on log-rank. ns, *P* > 0.05; *, *P *< 0.05; **, *P* < 0.01 (Mantel-Cox test).

To verify whether l-serine is associated with drug resistance in S. suis, we used the drug-resistant strain obtained by laboratory induction of ATCC 700794, which was resistant to macrolide antibiotics and had severalfold-higher MICs for tylosin, aivlosin tartrate, and tilmicosin than did the S. suis sensitive strain (Table 1). We also examined the toxic effects of l-serine on S. suis, and the results showed that the addition of 50 mM l-serine had no significant effect on the survival of either sensitive or resistant strains of S. suis (Fig. 2A). We then treated the bacteria with 1/2 MIC drug levels in the presence of different l-serine concentrations. The experimental results demonstrated that this potentiation occurred in an l-serine dose-dependent manner for tylosin (Fig. 2B). We observed that bacterial survival decreased upon treatment with 1/2 MIC or 1/4 MIC drug levels of tylosin, aivlosin tartrate, and tilmicosin in the presence of l-serine (Fig. 2C). In particular, the germicidal effect of 1/2 MIC of tylosin in combination with l-serine against S. suis resistant strains reached 99%, much higher than the 1% fungicidal effect observed after the action of single tylosin on the strain. Similarly, the combination of either tilmicosin or aivlosin tartrate with l-serine was able to achieve more than 90% bactericidal effect against the resistant strain. These data show that l-serine can enhance antibiotic susceptibility of drug-resistant strains of S. suis.

**TABLE 1:** MICs for Streptococcus suis in this study

Antimicrobial	S. suis sensitive strain MIC (mg/L)	Interpretation^*a*^	S. suis resistant strain MIC (mg/L)	Interpretation^*a*^
Tylosin	0.125	S	128	R
Tilmicosin	0.25	S	32	R
Tilmicosin concn	0.25	S	256	R

a Interpretation of antibiogram: S, sensitive (active substance normally effective against microorganisms at the recommended dosage); I, intermediate (active substance may be effective against microorganisms at higher than the recommended dose); R, resistant (active substance not effective against microorganism in either recommended or higher dosage due to resistance mechanism).

### The addition of l-serine can affect the formation of bacterial biofilms.

In clinical practice, the formation of bacterial biofilms is an important factor in infectious diseases. Therefore, it would be clinically useful to attenuate the ability of bacterial biofilms to form in the presence of l-serine. Biofilms of S. suis resistant strains were cultured *in vitro* in the presence or absence of l-serine to examine the effect of l-serine on biofilm formation (Fig. 2D). The results showed that macrolide-resistant strains had a significantly decreased ability to form biofilms after supplementation with l-serine.

### *In vivo* experiments analyzed the therapeutic effect of l-serine combined with tylosin.

A mouse thigh infection model was established to evaluate the synergistic effect of l-serine and tylosin against drug-resistant strains of S. suis *in vivo*. The change in viable bacterial counts in the mouse thighs was determined by colony counting. The results showed that after the combined treatment with serine and tylosin, the number of bacteria in the mouse thigh infection model was significantly reduced compared to that seen with treatment with either tylosin or l-serine alone (Fig. 2E). Meanwhile, it was found that l-serine and tylosin also had synergistic effects in an *in vivo* experiment establishing an acute infection model of Galleria mellonella. Specifically, only 10% of larvae survived when G. mellonella was infected with a resistant strain. When infected G. mellonella larvae were treated with either tylosin or l-serine alone, the survival rates were 40% and 20%, respectively. However, the survival rate of G. mellonella increased to 80% when treated with a combination of tylosin and l-serine (Fig. 2F). These results indicate that l-serine promotes tylosin activity to kill multidrug-resistant S. suis.

### l-Serine addition affects serine metabolic pathways.

**FIG 3:**
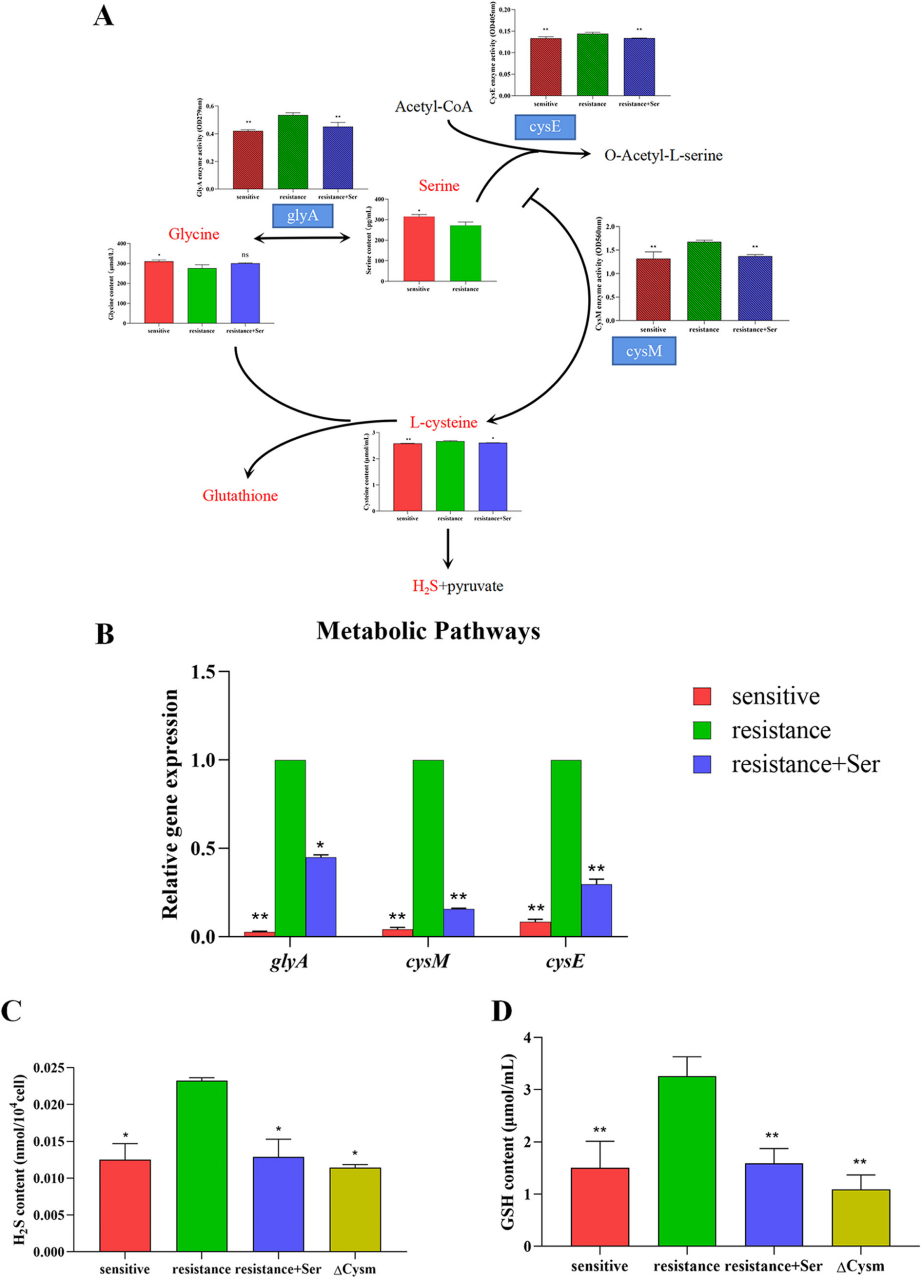
Metabolic pathway analysis. (A) Metabolite content and enzyme activity (shading) of the serine catabolic pathway in the resistant strain after addition of 50 mM l-serine, compared to that in the control sensitive strain. (B) The gene expression levels of the serine catabolic pathway of the resistant strain were examined via RT-PCR after exogenous addition of l-serine and compared to those of the control sensitive strain. (C and D) H_2_S (C) and GSH (D) contents in the resistant strain with exogenous addition of 50 mM l-serine and in the *cysM* gene mutant strain compared to that in the control sensitive strain. All data are means ± SD. *P* values were determined using an unpaired, two-tailed Student's *t* test. ns, *P *> 0.05; *, *P* < 0.05; **, *P* < 0.01.

To understand how the serine metabolic pathway is activated by l-serine, we further evaluated metabolite content, enzyme activity, and gene expression in the pathway. As shown in Fig. 3A, in the biphasic response of serine and glycine regulated by serine hydroxymethyltransferase (GlyA) in drug-resistant strains, as the amount of serine decreased, the amount of glycine also decreased, but the activity of GlyA was enhanced. At the same time, as the serine concentration decreased, the concentration of cysteine generated by the combined action of serine and oxyacetyl serine increased, owing to feedback inhibition. The activities of two important enzymes in the reaction, serine acetyltransferase (CysE) and cysteine synthase (CysM), were also significantly enhanced in the macrolide-resistant strain (Fig. 3A). Changes in *glyA*, *cysE*, and *cysM* gene expression followed patterns seen for changes in enzyme activity (Fig. 3B). Finally, the levels of the cell-protective metabolites H_2_S and glutathione (GSH), produced by glycine and cysteine as the main donors, were also significantly increased in the resistant strain compared to that in the sensitive strain (Fig. 3C and D). When l-serine was added for the S. suis resistant strain, the metabolite content, enzyme activity, and gene expression in the serine metabolic pathway were significantly altered.

### Enhancement of bacterial endogenous ROS by l-serine due to inhibition of H_2_S and GSH.

**FIG 4:**
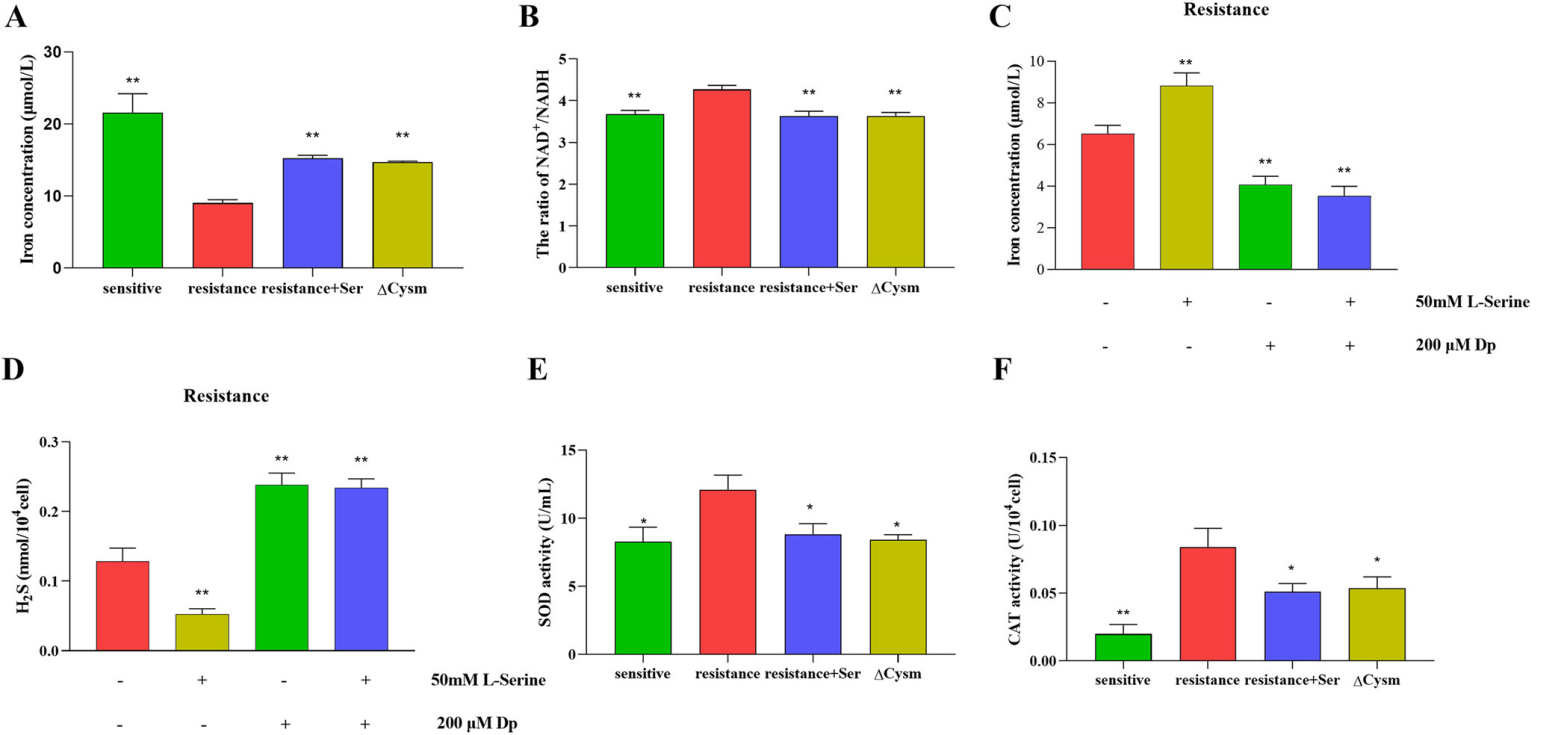
Effect of l-serine addition and *cysM* gene deletion on H_2_S and Fenton response in S. suis. (A and B) Iron (A) and NAD^+^/NADH (B) content of S. suis in the presence of 50 mM l-serine. (C and D) Effects of the addition of 200 μM iron-coupling agent DP on hydrogen sulfide (D) and iron (C) content in S. suis. (E and F) SOD (E) and CAT (F) enzyme activities of S. suis in the presence of 50 mM l-serine. All data are means ± SD. *P* values were determined using an unpaired, two-tailed Student's *t* test. ns, *P* > 0.05; *, *P* < 0.05; **, *P* < 0.01.

To investigate how S. suis acts through the serine catabolic pathway and the mechanism of action by which metabolites stimulate an increase in ROS after exposure to exogenous l-serine, we monitored H_2_S, GSH, and action-related enzyme activities of the resistant and Δ*cysM* strains treated with l-serine. The results showed that in the resistant strain, the iron concentration decreased significantly with increasing H_2_S content, whereas the iron concentration increased significantly after l-serine was added (Fig. 3C and 4A). At the same time, the downregulation of the NAD^+^/NADH ratio in the resistant strain after the addition of l-serine proved that the intracellular Fe-S cluster was disrupted, while endogenous iron was released (Fig. 4B). This implies that the development of resistant strains is due to the inhibition of the Fenton response and that l-serine alleviates this metabolism change. To further verify that the Fenton reaction was affected, we performed experiments using the iron chelator 2,2′-dipyridyl (DP). The results showed that in the presence of DP, the iron content of the resistant strain decreased significantly (Fig. 4C) and the H_2_S content increased significantly (Fig. 4D). The addition of DP for the resistant strain significantly restored the synthesis of H_2_S inhibited by l-serine (Fig. 4D). Similarly, DP alleviated the bactericidal effect of tylosin in combination with l-serine against the resistant strain (see Fig. S4). In contrast, ROS removal by superoxide dismutase (SOD) and catalase (CAT) was significantly enhanced by H_2_S. In contrast, when supplemented with l-serine, both CAT and SOD activities were significantly downregulated (Fig. 4E and F). The deletion of *cysM*, which regulates cysteine synthesis in resistant strains, was found to act similarly to the inhibition of cysteine synthesis in resistant strains upon the addition of l-serine, both of which promote a Fenton response and reduce the protective effect of the bacteria themselves through the release of iron.

**FIG 5:**
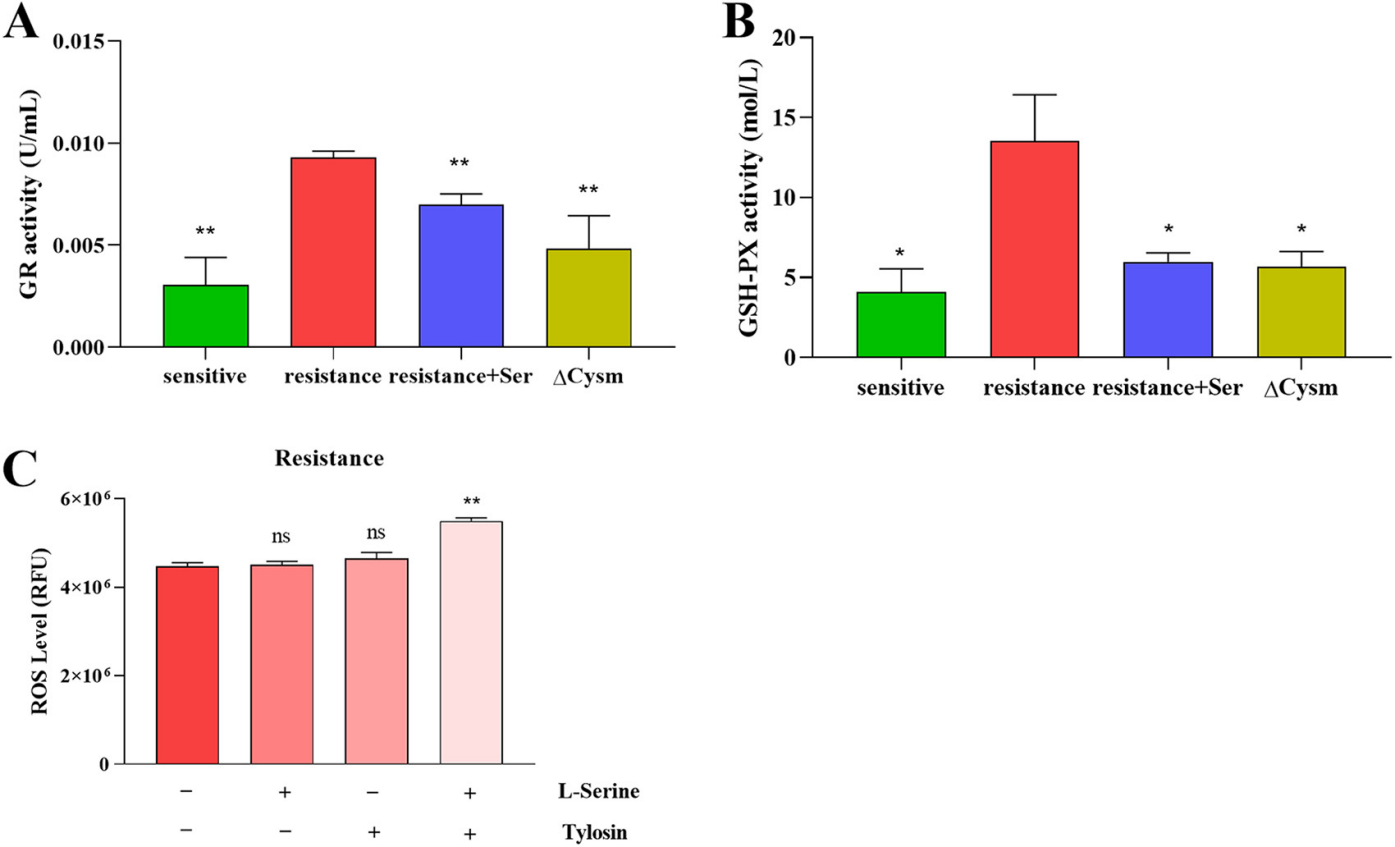
Effects of l-serine addition on GSH-related enzyme activity and ROS levels in S. suis. (A and B) GR (A) and GSH-PX (B) enzyme activities in the resistant strain with exogenous addition of 50 mM l-serine and mutant strains compared to that in the control sensitive strain. (C) Effect of 50 mM l-serine in combination with tylosin on ROS levels in resistant strains of S. suis. All data are means ± SD. *P* values were determined using an unpaired, two-tailed Student's *t* test. ns, *P *> 0.05; *, *P *< 0.05; **, *P* < 0.01.

GSH can protect bacteria from oxidation through binding to ROS in bacteria. Therefore, in addition to GSH, we examined the activity of GR (glutathione reductase) and GSH-PX (glutathione peroxidase), two important enzymes in the GSH redox reaction. The results showed that addition of l-serine significantly reduced the activity of GR and GSH-PX in the resistant strains (Fig. 5A and B). This demonstrated that the interconversion between reductive and oxidative GSH is weakened. Meanwhile, GSH-mediated protection against the threat of oxidative stress was inhibited in bacteria, which restored the resistant strain to a metabolic level similar to that of the sensitive strain. Deletion of the *cysM* gene and addition of l-serine had similar effects on the resistant strains.

### Addition of l-serine altered ROS levels.

To test whether l-serine can increase the production of intracellular ROS, ROS levels were compared after the addition of macrolides alone or in combination with l-serine, using 2′,7′-dichlorodihydrofluorescein diacetate (DCFH-DA). The results showed that there was a significant increase in ROS levels within S. suis after the combination of tylosin and l-serine (Fig. 5C). To determine whether an increase in ROS levels can affect bacterial survival, we measured the survival of an S. suis resistant strain treated with H_2_O_2_. The results showed that the inhibitory effect of H_2_O_2_ on S. suis was dose dependent, and 1 mM H_2_O_2_ already had a highly significant bactericidal effect (see Fig. S5A). Moreover, the addition of l-serine did not alter the bactericidal effect of H_2_O_2_ on S. suis (see Fig. S5B).

### Addition of l-serine enhances DNA damage in bacteria.

**FIG 6:**
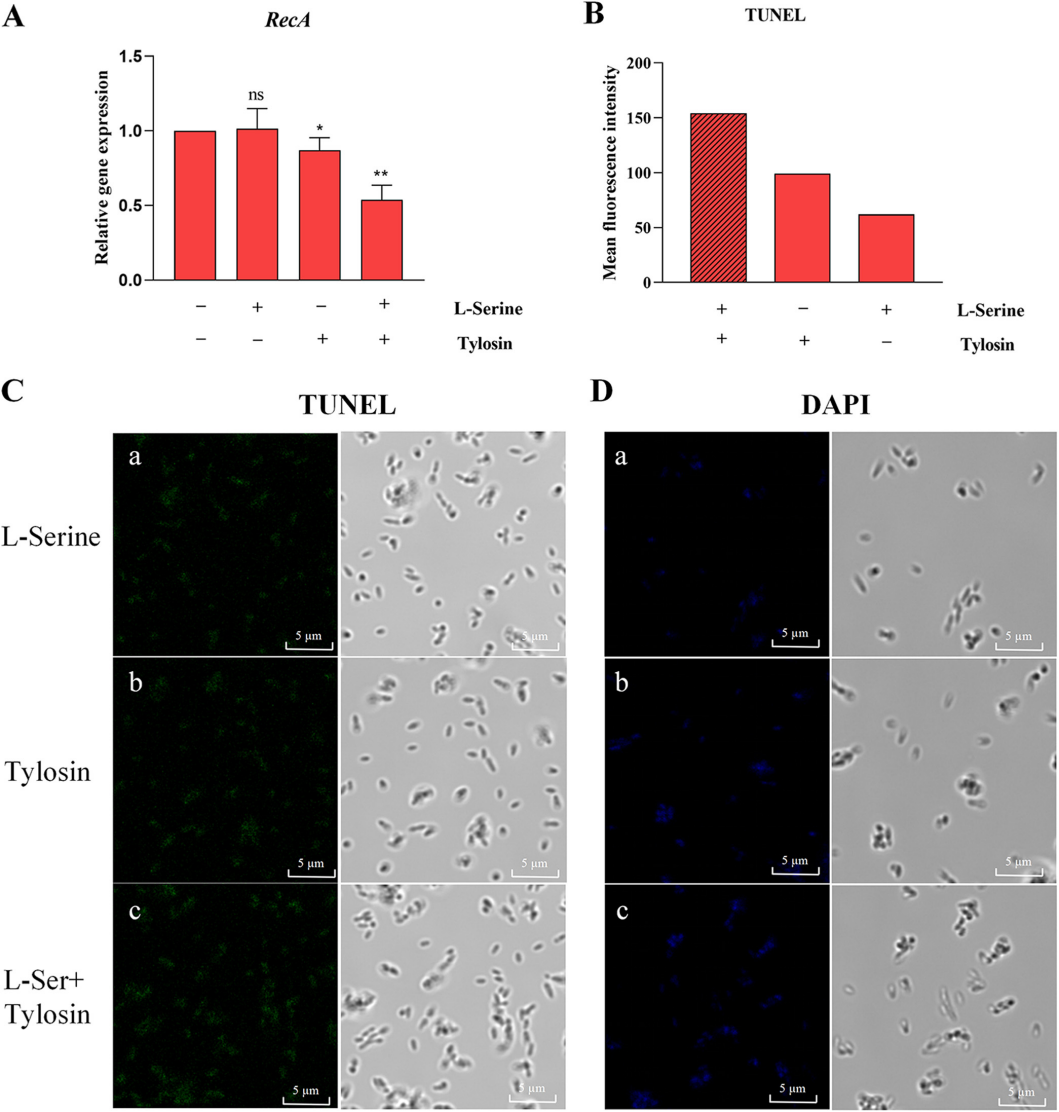
l-Serine enhanced the intensity of DNA damage in S. suis treated with tylosin. (A) The levels of *recA*, a key gene for the SOS response in the resistant strain, under tylosin treatment were examined by RT-PCR with and without addition of l-serine. All data are means ± SD. *P* values were determined using an unpaired, two-tailed Student's *t* test. ns, *P *> 0.05; *, *P *< 0.05; **, *P *< 0.01. (B) In the presence or absence of l-serine, double-stranded DNA breaks were detected in the resistant strain after tylosin action using a FACSCalibur flow cytometer with TUNEL fluorescence. (C and D) TUNEL (C) and DAPI (D) fluorescence was observed under a fluorescence microscope. (a) l-Serine-treated cells alone; (b) tylosin-treated cells alone; (c) cells treated with tylosin after exogenous addition of l-serine at 37°C for 3 h.

To study the alteration in the occurrence of DNA damage in bacteria by l-serine, we investigated its effect on the global response to DNA damage (SOS response). The SOS system involves the *recA* gene, which is stimulated by single-stranded DNA. We measured expression of the *recA* gene of an S. suis resistant strain in which l-serine and tylosin were cointeracting. The addition of l-serine significantly inhibited the expression of *recA* in the S. suis resistant strain (Fig. 6A). Staining of DNA double-strand breaks in cells using the terminal deoxynucleotidyltransferase-mediated dUTP-biotin nick end labeling (TUNEL) method revealed that there were significantly more DNA double-strand breaks in S. suis cells treated with the combination of l-serine and tylosin than in the groups treated with l-serine or tylosin alone (Fig. 6B and C).

4′,6-Diamidino-2-phenylindole dihydrochloride (DAPI) is used to label DNA in the chromosomes of coalescing cells (25). Compared to S. suis resistant strain cells without l-serine treatment, which showed only slight fluorescence, S. suis cells cotreated with l-serine and tylosin showed intense fluorescence, indicating the presence of condensed chromosomes in these cells (Fig. 6D). DAPI staining confirmed chromosome condensation, indicating replication arrest, and TUNEL staining demonstrated the appearance of DNA double-strand breaks. These results strongly suggest that l-serine triggers DNA damage in S. suis cells as a result of intracellular ROS accumulation.

## DISCUSSION

The threat of bacterial resistance forces a more effective response. The development of bacterial resistance has been shown to have an important relationship with physiological metabolism (24). To study the mechanism of macrolide resistance in S. suis and to find countermeasures, we performed metabolomic assays on macrolide-resistant S. suis and a standard strain of ATCC 700494 to analyze physiological metabolic differences. Our results showed that the glycine, serine, and threonine metabolic pathways were significantly affected. l-Serine had an important differential impact on metabolism in this pathway. It has been found that serine can make E. coli less tolerant to gentamicin (26). Therefore, we propose that the development of macrolide resistance in S. suis is also caused by the altered physiological metabolism of l-serine.

In this study, we found that the combination of l-serine and tylosin could enhance DNA damage in S. suis by increasing the production of ROS. The protective function of serine is most pronounced in drug-resistant strains, where defects in serine metabolism allow the overproduction of endogenous H_2_S and complete protection of cells from ROS toxicity and DNA damage (27, 28). As a unique small signal molecule driving redox reactions, H_2_S plays an important role in the electron transport chain (29) and the Fenton reaction (30). It has been shown that the Fenton reaction is restored when the conversion of cysteine to H_2_S is inhibited, allowing the release of free Fe^2+^ in bacteria (31). The Fenton reaction cleaves H_2_O_2_ in bacteria to generate highly toxic hydroxyl radicals, which in turn produce bactericidal effects (32, 33). In the present study, the addition of l-serine reduced the production of H_2_S by inhibiting the conversion of cysteine to H_2_S, leading to the onset of ROS-induced oxidative stress. Cysteine serves as the main sulfur and carbon source of H_2_S and GSH (34, 35), and its biosynthesis is regulated by the *cysM* gene (36). Therefore, we selected *cysM* gene deletion strains for validation. In fact, in drug-resistant strains, deletion of the *cysM* gene had a similar effect to the addition of l-serine, both of which inhibited H_2_S production. Both promote the occurrence of the toxic Fenton reaction that allows excessive ROS production and leads to bacterial death due to DNA damage (31).

In addition, l-serine metabolism affects GSH synthesis (37). The carbon in GSH is derived from both glycine and cysteine (38). GSH is the main cytosolic thiol that protects cells from oxidative stress, and it can form disulfide bonds with other sulfhydryl-containing proteins and participate in the reduction of disulfide bonds. It can also protect cells from oxidative stress by binding to ROS (39). In the presence of oxidative stress, GSH reduces H_2_O_2_ to H_2_O and oxidizes itself to an oxidized form (GSSG) in the presence of GSH-PX, whereas it reduces GSSG to GSH again in the presence of GR, which participates in intracellular redox reactions and consumes excess intracellular hydroxyl radicals (17). This is also an important mechanism by which ROS regulate protein function and cell signaling. In the present study, S. suis resistance to macrolides was followed by increased GSH levels and significantly enhanced GR and GSH-PX enzyme activity. In contrast, the addition of l-serine resulted in the inhibition of GSH synthesis, reduction of ROS bound to GSH, and restoration of bacterial ROS levels. The GSH content in the *cysM* deletion strain was determined, and the results were consistent with those obtained with the exogenous addition of serine. These results further demonstrated that serine inhibits the cysteine biosynthetic pathway, leading to a decrease in GSH levels.

When bacteria are exposed to peroxides, they are subjected to the action of toxic hydroxyl radicals generated by intracellular redox reactions, causing DNA double-strand breaks and bacterial death (30). The SOS response is an important stress response in bacteria as it is involved in the regulation of multiple cellular responses, and it is an important protective mechanism for bacteria in response to DNA damage (40). RecA and LexA regulate and participate in the SOS response, DNA replication, mutation and repair, protein synthesis, cell division, and many other life processes (41). Under stress conditions, *recA* is activated by binding to single-stranded DNA and assists in *lexA* induction to enable SOS responses (42). The SOS response to repair is regulated by *recA* and *lexA*, with implications for the cell cycle, cell division, and DNA replication (32). Bacteria grown under aerobic conditions are regulated by their own defense mechanisms and are protected from damage caused by ROS generated by respiration (43). However, when stress leads to excessive ROS generation, it can cause DNA damage and impaired replication, which can be harmful to bacteria (44). In the present study, we found that the effect of l-serine on drug resistance in S. suis was associated with ROS production and DNA damage-mediated oxidative stress. The modulation of the Fenton reaction and redox of GSH after the combination of l-serine with macrolides correlated with the increase in ROS content in bacteria. When the level of l-serine is reduced, the level of ROS is lowered, and the SOS response in bacteria is activated to repair DNA damage and keep the bacteria alive.

This suggests that alterations in the metabolic status play an important role in the development of bacterial drug resistance. In the TUNEL and DAPI staining experiments, an increase in DNA double-strand breaks and condensation of chromosomes, representing replication arrest induced by the combined action of l-serine and macrolides, demonstrated an enhanced effect of ROS-induced DNA damage. Thus, the development of macrolide resistance in S. suis is mainly due to the inhibition of ROS production by the l-serine metabolic pathway. ROS is a key substance of aerobic metabolism that can damage many macromolecules, such as DNA, proteins, and lipids (28). Furthermore, the SOS response is an important stress response in bacteria that is involved in the regulation of multiple cellular responses to DNA damage (40). In brief, l-serine was added to enhance the inhibitory effect of macrolide on drug-resistant S. suis by inducing DNA damage through modulation of ROS production, resulting in inhibition of the SOS response.

**FIG 7:**
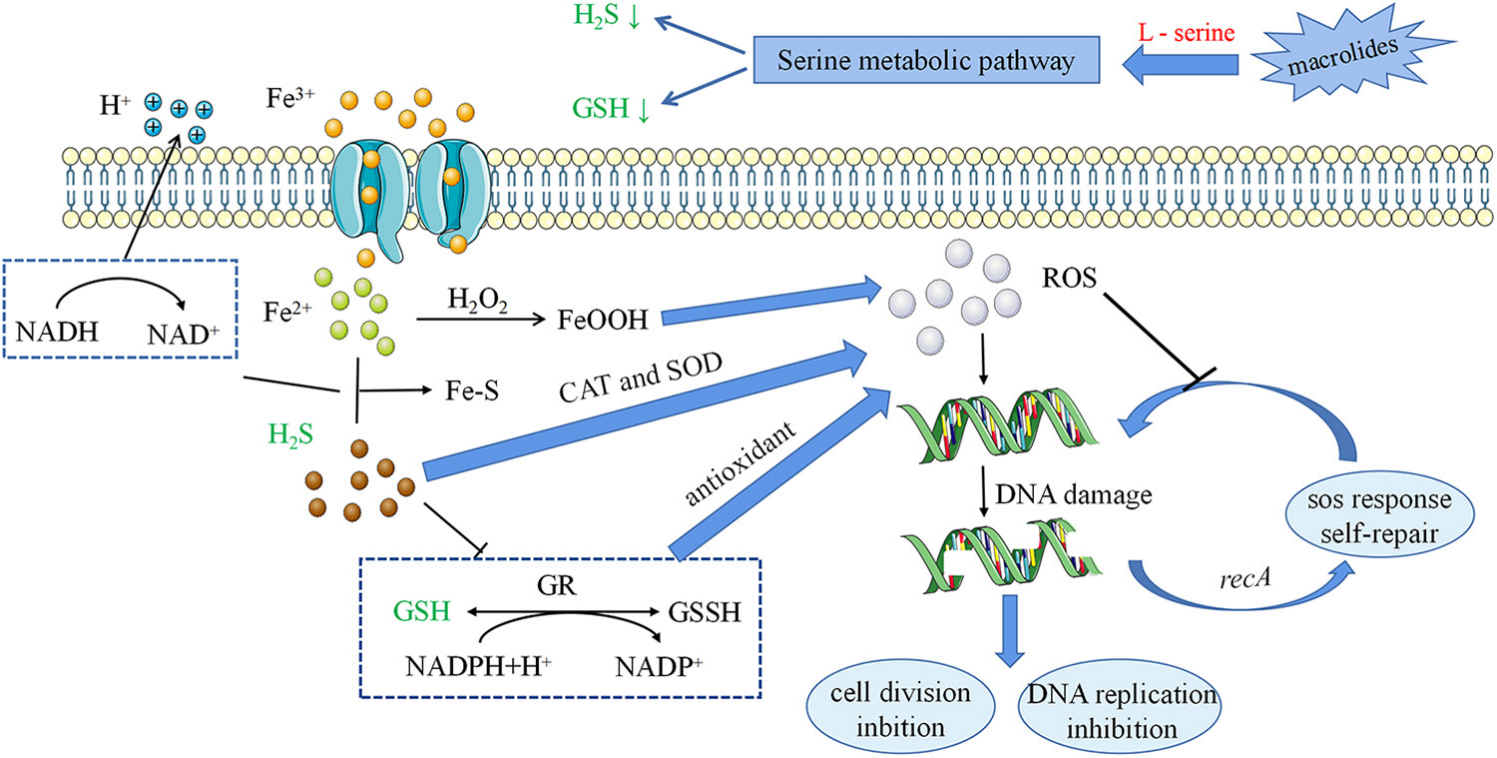
Effects of serine metabolic pathways and oxidative stress on the mechanism of action of resistant strains of S. suis.

Overall, these results suggested that the serine metabolic pathway is associated with the development of macrolide resistance in S. suis. Moreover, l-serine enhanced the antibacterial effects of macrolides against S. suis *in vitro* and *in vivo* by regulating ROS production (Fig. 7). Importantly, they revealed new insights into the mechanisms of macrolide resistance and expanded the targets that may lead to anti-infective treatment options.

## MATERIALS AND METHODS

### Strains and growth conditions.

S. suis ATCC 700794 used in this study was purchased from the American Type Culture Collection and stored in our laboratory. Unless otherwise stated, the bacteria were cultured in Todd-Hewitt broth (THB) at 37°C under aerated conditions. Strain ATCC 700794 was induced to have macrolide resistance levels by broth dilution under long-term selection pressure of tylosin *in vitro* (45) and designated the resistant strain. All experiments were performed using bacteria in the exponential phase. We used MICs to assess sensitivity to antibiotics.

### Antibiotics and chemicals.

Tylosin, aivlosin tartrate, tilmicosin, and l-serine were purchased from Solarbio (Beijing, China). l-Serine was dissolved in double-distilled H_2_O to a 500 mM stock solution. After filtering the stock solution with a sterile hydrophilic polyvinylidene difluoride membrane with a pore size of 0.22 μm (Biosharp, China), it was stored at −20°C. The solution was diluted according to the concentration required.

### MIC determinations.

MICs of tylosin, aivlosin tartrate, and tilmicosin were determined by the microdilution method in THB medium and then incubating the samples at 37°C for 24 h. The MIC was defined as the minimum antibiotic concentration that inhibited bacterial growth. All samples were tested at least three times for MIC determinations.

### Metabolome assay and data analysis.

For targeted metabolomic analysis, samples were precipitated and analyzed using a UHPLC 1290 Infinity instrument (Agilent Technologies, USA) and a coupled quadrupole time-of-flight mass spectrometer (AB TripleTOF 6600) equipped with an electrospray ionization source. Detection was performed in both negative and positive ion modes. The raw MS data were converted into MzXML files using a ProteoWizard MSConvert tool and processed using XCMS for feature detection, retention time correction, and alignment. The metabolites were identified by accuracy mass spectrometry (<25 ppm) and MS/MS data, which matched our standard database. For multivariate statistical analysis, the web-based MetaboAnalyst system (https://www.metaboanalyst.ca/) was used, followed by Pareto scaling, PCA, and OPLS-DA. The significantly different metabolites were determined based on the combination of a statistically significant threshold VIP value obtained from the OPLS-DA model and two-tailed Student’s *t* test (*P* value) on the raw data, and the metabolites with VIP values of >1.0 and *P* values of <0.05 were considered significant.

### Antibiotic survival assay.

To obtain exponential and mid-stationary phase cultures in THB, the 16-h culture was diluted 1:1,000 in fresh medium and grown to the desired turbidity (optical density at 600 nm [OD_600_] of 0.5). The cultures were collected and resuspended in THB medium. Different antibiotics (tylosin, aivlosin tartrate, and tilmicosin) at concentrations of 1/2 MIC and 1/4 MIC were added. l-Serine was incubated in the medium alone or simultaneously with the antimicrobial drug at the specified concentrations. S. suis incubation without any drug was used as a control group. After 24 h of incubation, the samples were removed for enumeration of colonies of S. suis. Bacterial survival was calculated as the ratio between the dosing and blank control groups. The results represent the averages of three biological replicates, and the error bars represent the standard deviations (SD).

### Biofilm assay.

Biofilm formation was quantified using the crystal violet staining method (46). Overnight cultures of S. suis sensitive and resistant strains were diluted 1:100 in fresh THB medium at a final concentration of 10^6^ CFU/mL and used to inoculate the wells of the microplates. The effect of l-serine addition on biofilm formation was also evaluated. The absorbance of each well was measured at 590 nm using a spectrophotometer. All assays were performed in triplicate.

### Thigh infection model experiment in mice.

A mouse thigh infection model (47) was used to assess the synergistic effect of the combination of l-serine and tylosin on S. suis. Six-week-old female specific-pathogen-free ICR mice were purchased from the Experimental Animal Center of the Second Affiliated Hospital of Harbin Medical University (Harbin, China). All animal experiments conformed to the ethical principles of animal research and were approved by the Institutional Animal Care and Use Committee of the Northeastern Agricultural University (NEAUEC20). The mice developed neutropenia (neutrophil count of <100 mm^3^) when cyclophosphamide was administered intraperitoneally at 150 and 100 mg/kg 4 days and 1 day before bacterial inoculation, respectively. The immunodeficient mice were divided into five groups for subsequent experiments (*n* = 5). Mice in the model group were injected intramuscularly with 100 μL of inoculum in each thigh, which included 10^7^ log-phase test strain. Two hours after modeling, mice were treated with tylosin (10 mg/kg) and l-serine (1 g/kg) alone or in combination as the tylosin single-treatment group, l-serine single-treatment group, and combination treatment group, respectively. Finally, the mice injected with physiological salt were used as blank controls. Both l-serine and tylosin were administered intramuscularly. After 22 h of drug treatment, mice were euthanized. Both posterior thigh muscles of the mice were immediately collected in normal saline, homogenized, and appropriately diluted, and the number of CFU was determined by planar colony counting. The thigh tissue CFU titer is reported as the log_10_ CFU per thigh.

### G. mellonella larvae.

G. mellonella larvae were purchased from Cade Ruixin International Trade Co., Ltd. (Tianjin, China) and stored in the dark at 15°C for use within 14 days. Larvae were separated by weight, and only those between 0.2 and 0.3 g were used in the experiment (48). The larvae of G. mellonella were randomly divided into six groups (*n* = 10 per group) and infected with 10 μL of S. suis resistant strain suspension (1.0 × 10^6^ CFU) at the right posterior gastropod. Two hours postinfection, G. mellonella was treated with l-serine (1 g/kg), tylosin (10 mg/kg), or a combination of tylosin plus l-serine (10 mg/kg plus 1 g/kg) in the left posterior gastropod. Each experiment included two negative controls: one group was not injected, to control mortality of background larvae (no-operation control), and the other group (uninfected controls) was injected with phosphate-buffered saline (PBS) to control for the possible effect of physical trauma on mortality. Larvae were kept in petri dishes at 37°C and 5% CO_2_ for a maximum of 120 h after injection, with survival checked every 24 h. Larvae were considered dead if they did not move after shaking the petri dishes. The survival rates of G. mellonella were recorded over 5 days.

### Metabolite concentration detection.

Levels of cysteine, glycine, and serine (Sinobestbio, China), and GSH and H_2_S (NanJing JianCheng Bioengineering Institute, China) were determined using content assay kits. The resistant strain was incubated in THB medium supplemented with l-serine for 12 h at 37°C, and the bacterial cultures were collected, washed, resuspended in PBS, and adjusted to an OD_600_ of 0.5. After sonicating and centrifuging 1 mL of the sample to break up the cells, the supernatant was collected and analyzed according to the kit’s instructions to detect the concentrations of metabolites in the bacteria. The assays were repeated three times.

### Measurement of enzyme activity.

SOD, CAT, GSH-PX, and GR levels were measured using colorimetric assay kits (NanJing JianCheng Bioengineering Institute, China). In brief, bacterial cultures were collected, washed, resuspended in lysis buffer (from the assay kits), and disrupted by sonication. After centrifugation, the supernatant was transferred to a new tube and reacted according to the manufacturer’s instructions, and absorbance was measured using an enzyme marker. The activity of the corresponding enzyme was calculated by creating a standard curve and substituting the absorbance value of the sample.

GlyA activity was determined according to the method of Jiang et al. (46). CysE activity was determined according to the method described by Qiu et al. (49). CysM activity was determined according to the method of Marie-Françoise Hullo et al. (50). The activity assays were repeated three times.

### Quantification of metabolism-related genes by RT-PCR.

Reverse transcription-PCR (RT-PCR) was used to analyze the relative expression of genes related to bacterial resistance and metabolism. Metabolism-related genes included *glyA*, *cysE*, and *cysM* in the serine metabolic pathway. The primer sequences used are listed in Table S1 in the supplemental material. The bacterial cultures were collected by centrifugation. Total RNA extraction (Omega, China) and cDNA synthesis (TaKaRa, China) were done according to the manufacturer instructions. The relative expression was normalized using the 16S rRNA gene as an endogenous control (45). The reaction conditions were 94°C for 10 min followed by 40 cycles of amplification at 94°C for 15 s and 60°C for 60 s (51). Two replicate analyses were performed for each sample, and expression data were collected from three biological replicate samples. All assays were performed in triplicate.

### Determination of iron concentrations.

Cellular iron levels were determined using the colorimetric ferrozine assay method described by JanRiemer et al. (52). Cultures of S. suis were incubated to an OD_600_ of 0.5 after the addition of l-serine or the iron chelator DP, and the cultures were incubated for 12 h and harvested by centrifugation. The cell pellet was washed twice with ice-cold PBS and resuspended in 1 mL 50 mM NaOH. The lysate was mixed with 10 mM HCl and an iron release reagent and incubated at 60°C for 2 h to quantify the bound iron. After the mixture was cooled to 25°C, 30 mL of the iron detection reagent was added. After 30 min, the absorbance of the samples was measured at 550 nm.

### NADH measurements.

S. suis culture was diluted to an OD_600_ of 0.5 and incubated with amino acids at 37°C for 12 h. The cell pellets were washed with PBS and resuspended in NADH extraction buffer. The samples were fragmented by sonication. Heat extracts were performed at 60°C for 30 min to decompose the NAD^+^ extraction buffer that was added to neutralize the extracts. The cells were centrifuged and the supernatant was collected using the enzy chrom NAD^+^/NADH assay kit (Beyotime Biotechnology, China).

### ROS determination.

ROS were detected with a reactive oxygen species assay kit based on DCFH-DA (Beyotime Biotechnology, China). The samples were incubated with 10 mM DCFH-DA. After washing, cells were treated with tylosin in the presence or absence of 50 mM l-serine. Samples were obtained at the indicated time points and used to monitor fluorescence intensity at an excitation wavelength of 488 nm and an emission wavelength of 525 nm.

### TUNEL assay.

For the TUNEL assay, cells were grown to an OD_600_ of 0.5, and aliquots were treated with 50 mM l-serine and tylosin for 4 h. The cells were fixed and labeled using the one-step TUNEL apoptosis assay kit (Beyotime Biotechnology, China). After the labeling reaction was stopped, the cells were washed three times with PBS and analyzed by flow cytometry using a FACSCalibur.

### DAPI assay.

DNA damage was analyzed using DAPI (Sigma, USA) staining. Cells were grown to an OD_600_ of 0.5, and aliquots were treated with 50 mM l-serine and tylosin for 4 h. The cells were then incubated with 1 mg/L DAPI in the dark for 10 min and washed three times with PBS. The cells were observed under a fluorescence microscope.

### Statistical analysis.

Data were collected for statistical analysis, and Student’s *t* test was conducted using SPSS version 26.0 software. Values were reported as means ± standard deviations. Growth curves were fit using GraphPad Prism 8. The different levels of statistical significance were set as follows: ns, *P *> 0.05; *, *P *< 0.05; **, *P* < 0.01.

### Data availability.

The data sets used and/or analyzed here are available from the corresponding author upon reasonable request. Metabolomic data were deposited in the MetaboLights database using the identifier MTBLS4618.
