# Supplementary material for: Effects of l-Serine on Macrolide Resistance in Streptococcus suis
Source: Microbiol Spectr. 2022 Jul 18;10(4):e00689-22. doi: 10.1128/spectrum.00689-22 (PMC9430912; doi:10.1128/spectrum.00689-22)
Supplement: Supplemental file 2 — Fig. S1 to S5. Download spectrum.00689-22-s0002.pdf, PDF file, 0.4 MB [file spectrum.00689-22-s0002.pdf]

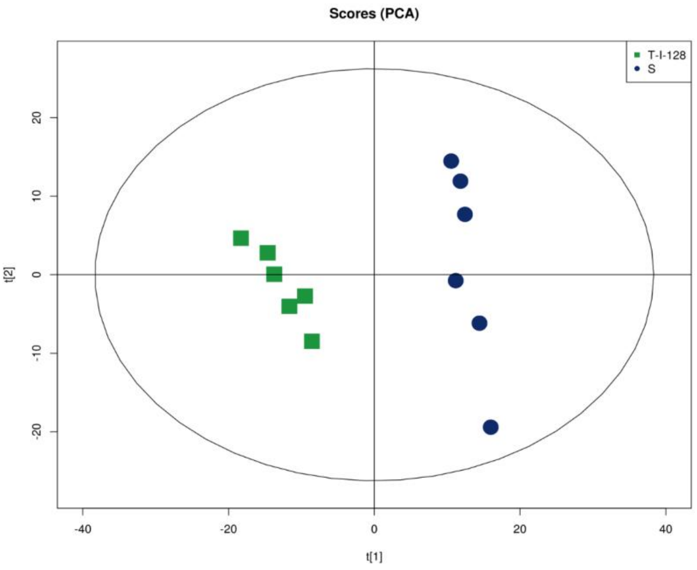

Fig. S1. Differential metabolisms of drug resistant (T-I-128) and sensitive strains (S) were analyzed for model reliability using unsupervised principal component analysis (PCA) modeling.

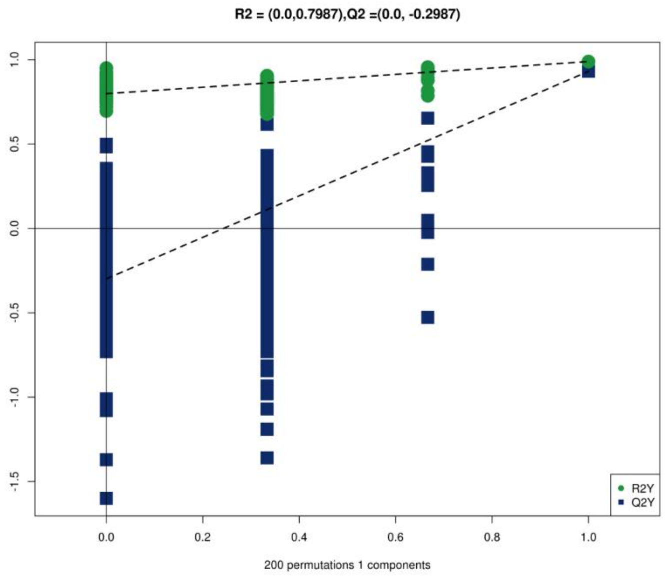

Fig. S2. The relationship between metabolite expression and drug resistant (T-I-128) and sensitive strain (S) was modeled using orthogonal partial least squares discriminant analysis (OPLS-DA). The model was used to predict the sample categories.

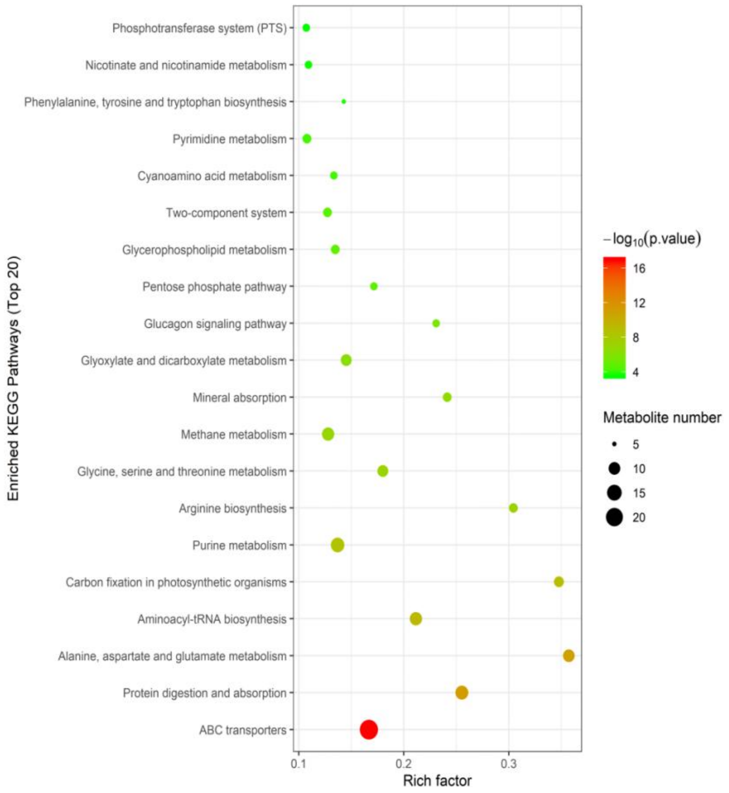

Fig. S3. Results of KEGG pathway enrichment analysis.

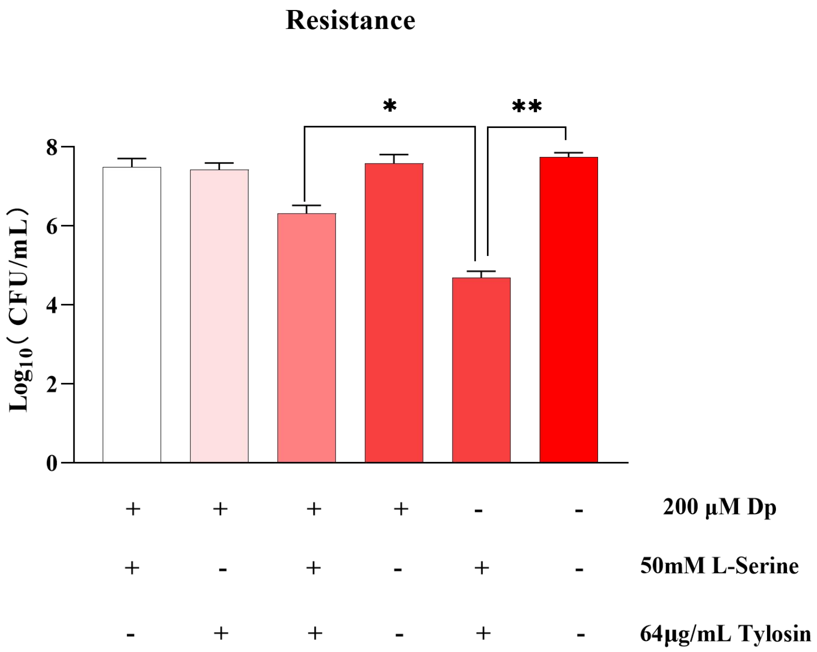

Fig. S4. Synergistic effect of L-serine and tylosin on resistant strains of *S. suis* after 200 µM iron coupling agent 2,2'-dipyridyl (dp) addition. All data are mean ± s.d. *p values* were determined using an unpaired, two-tailed Student's *t*-test. ns, $p > 0.05$; *, $p < 0.05$; **, $p < 0.01$.

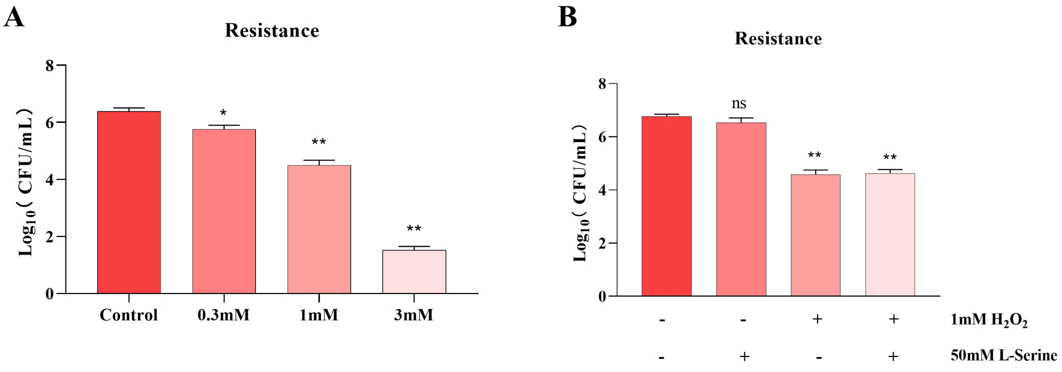

Fig. S5. Effect of H_2O_2 addition on the survival of resistant strains of *S. suis.* (A) Effect of 0.3 mM, 1 mM, 3 mM H_2O_2 addition on the survival of resistant strains of *S. suis*. Control is a control group without H_2O_2 addition. (B) Effect of 1 mM H_2O_2 on the survival of resistant strains of *S. suis* after the addition of 50 mM L-serine. All data are mean ± s.d. *p values* were determined using an unpaired, two-tailed Student's *t*-test. ns, $p > 0.05$; *, $p < 0.05$; **, $p < 0.01$.
